# Cold Blows the Wind

**Published:** 1856-01

**Authors:** 


### COLD BLOWS THE WIND

This January night, chilling the heart's blood of many a toiling widow, of many an orphan or more than orphan child, in this multitudinous city, in its damp and noisome basements, in its dark and cheerless garrets, its filthy alleys and its reeking lanes. Have a thought for them, you more favored few! who this blessed hour revel in happiness, well warmed, well clothed, well fed, gathering around your parlor fires, surrounded by wife, children and friends. Stop the music, hush the laughter, think and make note of it, how many a garment thrown aside to be worn no more, lies useless in the many lockers of your mansion, then bid the music and the mirth go on: it will be sweeter than before. But to-morrow send those rejected dresses, coats, shoes, &c. to No. 11 Clinton Hall, Astor Place, New York; and if you please, a few dollars cash inside the box. Send them, freight-paid, by some city express. Friend Crafts says who lives at 130 East Twenty-first street, or office 74 Maiden Lane, for a shilling he will take a good sized box there, where will be found a gentleman of talents and standing, and who has a heart just large enough to induce him to spend his whole time in finding out, not where such things are needed simply, but where they may be worthily and judiciously bestowed. You need not send your name, "*its record is on high.*"

